# Comparison of post-auricular and frontal bispectral index values obtained during renal surgeries

**DOI:** 10.1186/s12871-023-02372-x

**Published:** 2023-12-19

**Authors:** Ahmed Mohammed Fetouh Abdelrahman, Amr Arafa Elbadry, Amany Faheem Omara

**Affiliations:** Anesthesiology, Surgical Intensive Care and Pain Medicine Department, Faculty of Medicine, Tanta University, Tanta, Egypt

**Keywords:** Bispectral index, Depth of anesthesia, Frontal, Post-auricular.

## Abstract

**Background:**

The bispectral index (BIS) monitor is one of the EEG-derived monitoring techniques and well-established devices used to measure the depth of anesthesia. This study aimed to assess the agreement of BIS values based on the positions of either post-auricular or frontal sensors in individual patients undergoing renal surgeries while lateral positions at various stages of anesthesia.

**Patients and methods:**

12 patients older than 18 years, ASA I-III patients scheduled for elective renal operations, two BIS were placed on each patient, one on each side of the post-auricular region and one across the forehead, and each sensor was connected to a different BIS monitor. We gathered three pieces of data at each of the six-time points: BIS score, signal quality index (SQI) score calculating the signal’s strength and electromyography (EMG) score: before the onset of anesthesia (awake) when the eyelash reflex is lost (LOC), after intubation (intubation), following the initial surgical incision, each 30 min throughout the procedure (maintenance), and at the moment the patient’s eyes open naturally after waking up from anesthesia (emergence).

**Results:**

The overall BIS value at the frontal position was significantly higher than the post-auricular position (52.5 ± 22.2 and 52.1 ± 22.1, respectively, *P* = 0.010). On the other hand, the BIS value was comparable between the frontal and post-auricular positions at LOC, intubation, 60, 120, and 80 min and at emergence. A strong link between the two sensor positions, as indicated by the correlation coefficient (r = 0.607, *P* < 0.001), and the Bland-Altman analysis revealed a small mean difference (-1.8) and a low (9.0/- 12.5) limit of agreement, with just 4.3% of the readings falling outside of it during the anesthetic maintenance period.

**Conclusion:**

Acceptable variation in BIS data was observed when obtained from the two different sensor positions for clinical usage. The post-auricular BIS sensor system may be a suitable substitute for an impractical frontal setup.

**Protocol Registration:**

The study was registered in clinicaltrials.gov on 11/07/2022 (trial registration number: NCT05451823).

## Introduction

The level of anesthesia can be tracked by a complete 16-lead, 8-channel electroencephalogram (EEG), which records the electrical activity and potentials in the cerebral cortex. EEG activity can be generically classified into four wave patterns: alpha, beta, theta, and delta. It primarily occurs at frequencies between 1 and 30 Hz. When a patient is resting with eyes closed, they produce alpha waves, which have a frequency of 8–14 Hz. Highly alert and focused patients will have beta waves, which have a frequency between 14 and 30 Hz. Theta waves, which have a frequency of 4–8 Hz, are present during “light” anesthesia or the early stages of sleep. At a frequency of 0.5 4 Hz, delta waves are present during “deep” anesthesia or deep sleep [1].

During general anesthesia, monitors obtained from electroencephalograms (EEG) allow the titration and maintaining a sufficient level of anesthesia, benefit from shortening the recovery period after awakening, and advantages from lowering the risk of anesthetic adverse events [2].

The bispectral index (BIS) monitor is one of the EEG-derived monitoring techniques and well-established devices used to measure the depth of anesthesia. It is a tool for quantitative EEG that is frequently utilized to evaluate the hypnotic effect of anesthesia. An acceptable level of the hypnotic state is recommended to be between 40 and 60 [3].

However, in prone and lateral positions as in renal surgeries, problems in BIS monitoring may occur. Other areas of BIS electrode placement besides those recommended by the manufacturer should be considered, such as the mastoid and post-auricular region. But the validity and reliability of their BIS values have been questioned [4].

However, given how close the forehead sensor is to the surgical site, using BIS during some surgeries can be difficult. Due to blood or an antiseptic cleaning solution contaminating the forehead sensor, there is a strong likelihood that BIS recording may be interrupted. The size and shape of a BIS forehead sensor in the shape of a long strip can significantly affect where the surgical incision will be made [5].

Numerous alternative BIS sensor positions have been investigated where the frontal configuration is impractical [4–8]. However, little research has looked at utilizing the BIS sensor to measure the depth of anesthesia at the post-auricular region.

Moreover, growing research observed a shift in alpha oscillations (7.5–12.5 Hz activity) from occipital brain regions toward anterior brain during wakefulness to GA (“alpha anteriorization”) [9].

This study’s primary goal was to determine the degree to which BIS values varied depending on the placements of either post-auricular or frontal sensors in patients undergoing renal surgeries while in lateral positions at different stages of anesthesia. Post-auricular and frontal BIS values were our primary outcome.

## Patients and methods

This prospective cross-sectional study was performed at Tanta University Hospitals, Egypt from July 2022 to January 2023 on 12 patients older than 18, American society of anesthesiologists (ASA) I-III patients who planned for elective kidney surgeries. The study obtained approval from Tanta University ethical committee unit in June 2022 (ID:35,561/6/22) and registered at clinicaltrials.gov on 11/07/2022 (trial registration number: NCT05451823).

The use of electrodes (BISTM Quatro Sensors, Aspect Medical Systems, Newton, MA, USA) over the forehead and post-auricular area is contraindicated in patients with incapacitating central nervous system or cerebrovascular disease, those who take psychiatric drugs now, those who have had neurosurgical intervention in the past, and those who have certain skin conditions, for example. These patients were excluded from the study. The patients gave their signed consent after being fully informed. To preserve participant privacy and data confidentiality, each patient received a description of the study’s goals, received a secret code number, and had images taken exclusively of the body parts related to the study. Standard monitoring was carried out as soon as the patients entered the operating room, including taking their temperature, blood pressure, electrocardiogram, oxygen saturation, end-tidal carbon dioxide tension, and inspired oxygen tension.

An Infinity bispectral index (BISx SmartPod®) was utilized to monitor the level of anesthetic. (Aspect Medical Systems, Newton, MA, USA) and an Infinity® Delta XL monitor (Dräger Medical, Lübeck, Germany). The Joint Commission on Accreditation of Healthcare Organizations (JCAHO), the American Heart Association, and the Emergency Care Research Institute all set guidelines for this device (American Hospital Association). The forehead and post-auricular region each received a standard BISx Quatro® Sensor from Aspect Medical Systems. Two BIS sensors (BISTM Quatro Sensors, Aspect Medical Systems, Newton, MA, USA) were applied to each patient before the induction of anesthesia. One sensor was placed across the forehead, and the other along the post-auricular area (BIS-VistaTM monitors, Aspect Medical Systems, Newton, MA, USA).

Patients were told to maintain a relaxed expression throughout induction (eyes closed, mouth closed, no facial expressions).

After cleaning the forehead skin with an alcohol swab that contained 70% alcohol, the sensor leads were subjected to digital pressure for 2 to 5 s. Wet gel electrodes that are disposable make up the sensor. Lead 4, the ground electrode, also measures the electromyographic activity of the frontalis muscle.

**Fig. 1:**
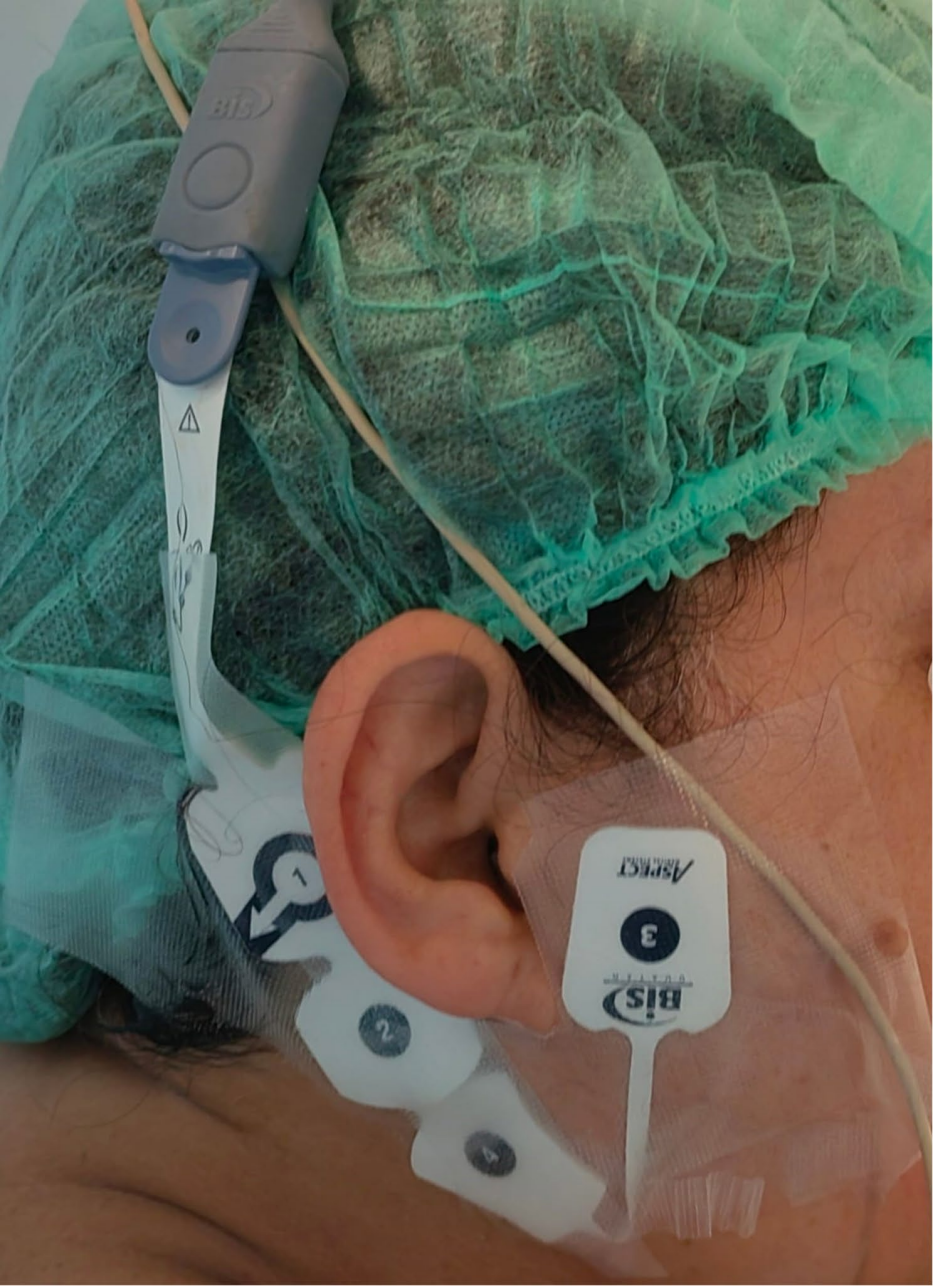
Location of bispectral index (BIS) sensors

Lead 1 of the frontal sensors was placed in the middle of the forehead, lead 2 was 2.8 cm to the left of lead 1, and lead 3 was placed in the temporal region between the lateral canthus and the hairline. Lead 1 of the post-auricular sensors was placed post-auricularly adjacent to the hairline, 2.5 cm medial to the mastoid area, on the same side of the face. The mastoid area was where lead 2 was placed, and the temporal region between the lateral canthus and the hairline was where lead 3 was attached on the same side. The BIS values between 40 and 60 were utilized to sustain the anesthetic depth titration (Fig. 1) [10].

We gathered three pieces of data in each case at each of the six-time points: BIS score, signal quality index (SQI) score calculating the signal’s strength and EMG score: before the onset of anesthesia (awake), when the eyelash reflex is lost (LOC), after intubation (intubation), after the first surgical incision (incision), every 30 min during the procedure (maintenance), and at the moment the patient’s eyes open naturally after waking up from anesthesia (emergence).

The data analysis removed the BIS values connected with sudden, high electromyography (EMG) ratings because they were considered artifacts.

### Statistical analysis

Considering the results of the earlier research by Akavipat and his associates [4], electrodes in the post-auricular area and the frontal region had a 0.74 correlation coefficient. According to the calculator online (http://sample-size.net/), a minimal sample size of 12 cases was necessary to attain an 80% power at a 0.05 significance level. After each procedure, data was collected from the BIS device for further examination. The Bland and Altman approach, regarded as the best methodology for comparing measurement modalities, was employed to contrast BIS data from the conventional frontal and post-auricular montage. Each patient’s individual data set and the entire data set were subjected to this analysis. To identify cases of unsuitable treatment alteration or failure to modify the treatment, when necessary, based on the standard montage score, we lastly looked at the data at or near the therapeutic limit of 60.

The study used 80% power and 95% significance levels. Statistical Package for Social Sciences (SPSS) version 26 for Windows was utilized to code, process, and analyze the collected data (SPSS Inc, Chicago, IL, USA). [11, 12] Numbers (frequency) and percentages, mean values, and standard deviations (SD), or a median and range were employed to display qualitative data. Two independent groups of qualitative data were compared utilizing the Chi-Square test (also known as Fisher’s exact test) for data comparison. For quantitative data, independent-Samples t-test and Mann-Whitney U test were employed to contrast two parametric and non-parametric data sets. It was statistically significant at *P* < 0.05.

A match-paired t-test was employed to contrast the means of BIS values across each time point. Statistical significance was assigned to the results if the p-value was less than 0.05. Pearson’s correlation and Bland-Altman analysis were performed using Med Calc software for Windows 8.1.0.0 (June 13, 2005). The results were displayed in a Bland Altman plot along with the bias and 95% limit of agreement. Clinically, the acceptable bias ranged from − 5 to 5. The IBM SPSS software program version 20.0 was utilized to examine the data fed into the computer. (Armonk, NY: IBM Corp). The Shapiro-Wilk test was employed to determine the normality of continuous data. For regularly distributed quantitative variables, range (minimum and maximum), mean, and standard deviation were employed to convey quantitative data. Frontal and Post-A were compared utilizing a paired t-test. The significance of the outcomes was assessed at the 5% level.

## Results

The mean age of studied cases was 56 ± 17.05. There was 4 (33.33%) male, 8 (66.67%) female. The mean BMI of studied cases was 30.27 ± 4.78 (Table 1).

**Table 1:** Descriptive analysis of the studied cases according to demographic data (n = 12)

		Mean ± SD
**Age (years)**		56 ± 17.05
**Sex**	**Male**	4 (33.33%)
	**Female**	8 (66.67%)
**BMI (kg/m** ^**2**^ **)**		30.27 ± 4.78

SD: Standard deviation

SQI and EMG were significantly higher in frontal than at post-A all measurements (*P* < 0.001) (Tables 2 and 3).

**Table 2:** Comparison between Frontal and Post-A according to SQI (n = 12)

	SQI		
Time	Frontal	Post-A	*p*
**Awake**	95.8 ± 4.4	86.2 ± 4.0	< 0.001^*^
**LOC**	95.0 ± 5.0	85.4 ± 4.4	< 0.001^*^
**Intubation**	92.0 ± 4.3	82.8 ± 4.0	< 0.001^*^
**Incision**	91.3 ± 4.4	82.1 ± 3.9	< 0.001^*^
**30**	91.7 ± 4.7	82.4 ± 4.1	< 0.001^*^
**60**	88.8 ± 3.8	80.0 ± 3.4	< 0.001^*^
**90**	90.2 ± 4.5	81.3 ± 4.1	< 0.001^*^
**120**	90.3 ± 4.3	81.3 ± 3.9	< 0.001^*^
**180**	93.3 ± 6.1	84.0 ± 5.6	0.001^*^
**Emergence**	92.3 ± 4.4	83.3 ± 4.0	< 0.001^*^
**Overall**	92.0 ± 4.8	82.8 ± 4.3	< 0.001^*^

SQI; signal quality index, LOC; the eyelash reflex is lost

**Table 3:** Comparison between Frontal and Post-A according to EMG (n = 12)

	EMG		
Time	Frontal	Post-A	*p*
**Awake**	34.67 ± 5.46	31.33 ± 5.79	< 0.001*
**LOC**	27.5 ± 1.05	25 ± 0.95	< 0.001*
**Intubation**	27.04 ± 0.99	24.58 ± 0.9	< 0.001*
**Incision**	27.32 ± 2.09	24.83 ± 1.9	< 0.001*
**30**	27.59 ± 2.32	25.08 ± 2.11	< 0.001*
**60**	26.95 ± 2.02	24.5 ± 1.83	< 0.001*
**90**	28.23 ± 1.95	25.67 ± 1.78	< 0.001*
**120**	26.51 ± 3.04	24.45 ± 2.88	< 0.001*
**180**	28.23 ± 2.29	25.67 ± 2.08	< 0.001*
**Emergence**	38.25 ± 4.65	35.5 ± 4.98	< 0.001*
**Overall**	26.42 ± 1.13	24.12 ± 1.09	< 0.001*

EMG; electromyography, LOC; the eyelash reflex is lost

The overall BIS value at the frontal position was significantly higher than the post-auricular position at awake, incision, 30 and 90 min (*p* < 0.05) and was comparable between the frontal and post-auricular positions at LOC moment, Intubation, 60, 120, and 180 min and at emergence (Table 4).

**Table 4:** Comparison between Frontal and Post-A according to BIS (n = 12)

	BIS		
Time	Frontal	Post-A	p
**Awake**	94.7 ± 5.3	93.8 ± 4.9	0.049*
**LOC**	24.2 ± 4.8	25.0 ± 4.4	0.054
**Intubation**	49.5 ± 8.8	49.3 ± 8.3	0.651
**Incision**	40.5 ± 9.0	39.7 ± 8.9	0.010*
**30**	44.4 ± 4.1	43.2 ± 3.3	**0.017** ^*^
**60**	43.6 ± 4.9	43.0 ± 4.2	0.171
**90**	42.2 ± 6.2	41.3 ± 6.3	0.014*
**120**	49.0 ± 6.3	48.9 ± 6.0	0.864
**180**	48.3 ± 4.5	47.7 ± 2.9	0.667
**Emergence**	85.3 ± 8.7	85.8 ± 7.7	0.546
**Overall**	52.5 ± 22.2	52.1 ± 22.1	0.010*

BIS; The bispectral index, LOC; the eyelash reflex is lost

**Fig. 2:**
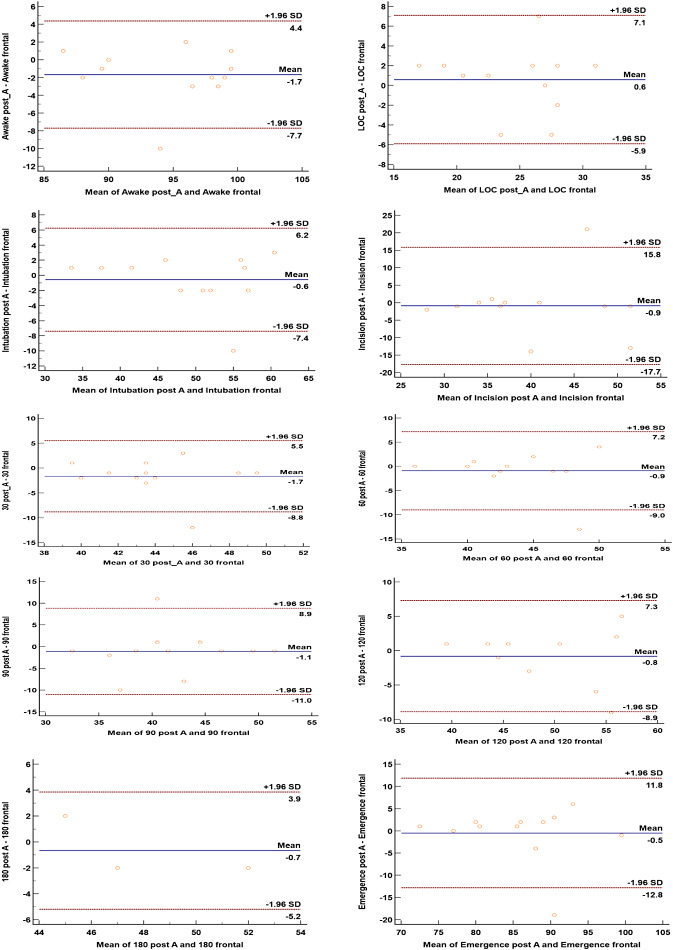
Bland Altman for BIS from Frontal and Post auricular in overall time

A strong link between the two sensor positions, as indicated by the correlation coefficient (r = 0.607, *P* < 0.001), and the Bland-Altman analysis revealed a small mean difference and a low limit of agreement, with just 4.3% of the readings falling outside of it during the anesthetic maintenance period (Fig. 2). Only 4.3% of the values during the anesthetic maintenance period fell outside the limit of agreement, according to the Bland Altman analysis of the correlation coefficient between the two sensor positions (r = 0.607, *P* < 0.001), which also revealed a strong relationship between the sensors and a low mean difference (-1.8) and a low limit of agreement (9.0/- 12.5). (Fig. 2)

## Discussion

When the sensor’s placement is in the surgical field, the manufacturer does not recommend the standard sensor position for BIS-guided anesthesia [13]. The accuracy of changing positions and applying alternate positions for monitoring anesthetic depth has been the subject of numerous research over the past years, with varying degrees of success [4–6, 8, 14–18].

The BIS sensor’s post-auricular position has been described and studied by Akavipat and colleagues [4]. Following the hairline with the last channel at the temporal region, the authors positioned the sensor 2.5 cm medial to the mastoid area. Their investigation deemed the discrepancy between the BIS values acquired from the forehead and post-auricular area acceptable.

In Dubey et al. study, BIS sensor placement at the supralabial site can be used as an alternative to the frontal placement in scenarios where the frontal position is the surgical site or is inaccessible during the maintenance of general anesthesia as in neurosurgery with particular emphasis on skin preparation and proper positioning of BIS electrodes to improve the signal quality [19]. Also, Akavipat et al. concluded that the post-auricular placement of a BIS electrode is a practical alternative to frontal lobe placement. Nevertheless, proper electrode location is important to minimize error [4].

In our investigation, to reduce the risk of positioning artifacts and achieve the best recordings, we chose to have a stronger concordance and safety monitoring of the depth of anesthesia when employing two sensors in the same patient undergoing lateral decubitus procedures. Overall, the alternate post-auricular posture and the traditional frontal position do not always meet BIS values. Although there was a significant correlation between the positions of the two sensors (r = 0.607), the Bland-Altman analysis showed a little mean difference (-1.8), and only 4.3% of the readings over the anesthetic maintenance period were outside the range of agreement, which is a significantly low limit of agreement (9.0/- 12.5). It may be clinically appropriate based on clinical values and correlation. Therefore, if the operating field prevents using the normal position during the anesthetic maintenance phase, it is possible to employ the post-auricular position as a backup. SQI evaluates the EEG signal’s quality after acquisition (0-100%). SQI is greater than 80, ensuring high-quality data [20]. During the waking, LOC, intubation, maintenance, and emergence periods, mean SQI values were considerably greater in the frontal sensor than in the post-auricular sensor position and significantly lower in the latter. However, we kept the mean SQI over 80 in both sensor positions. This result indicates the reliability of the post-auricular BIS readings.

EEG artifacts caused by facial muscle EMG activity can significantly lower the BIS score [21]. The anesthetic’s state has a significant impact on EMG ratings. In contrast to the frontal position, the mean EMG score was lower in the post-auricular position. This might be because human post-auricular muscles are less active than human forehead muscles. Following the muscle relaxant’s administration, the difference became less noticeable. This is another reason post-auricular BIS scores were lower overall than frontal BIS scores. The mean EMG was lower than 30 during anesthesia in both sensor placements. Thus, this demonstrates the accuracy of the BIS readings. Additionally, earlier research by Nelson and Puente-Barbas [5, 14] studied the potential for successful sensor placement at the nasal bridge and the infra-orbital region.

Additionally, much research looked at the sensor’s location in the occipital position. A case study by Hemmerling and associates [15] showed that the values between the commercially advised location and the occipital BIS sensor position were agreed upon. Similar outcomes were reported in a case study by Sinha and associates [16] utilizing spectral entropy. Shiraishi’s latest research was published [17]. On 25 patients, a strong association between frontal and occipital BIS values was found, and units were utilized as the acceptance threshold.

Another study by Brown et al. [22] collected 1812 paired readings from 16 patients. Showed 95% limits of agreement ranged between − 17.6 and + 33.1 and a 0.8% incidence of potential awareness (BIS > 60) measured by the frontotemporal approach which was not picked up by the auricular approach. They demonstrate that the limits of agreement are too wide for the auricular approach to be used in substitution of the frontotemporal approach. Using the auricular approach not only increases the risk of not detecting awareness, but also under-estimates the depth of anesthesia by a larger margin. This could potentially lead to unnecessarily increasing the depth of anesthesia, then increased risk of morbidity and mortality [22].

This study is limited by relatively small sample size and being a single center study. Further studies are needed in different types of surgeries.

## Conclusion

Based on the results of our study, it can be suggested that the post-auricular sensor position is a viable alternative to the conventional sensor position.

## Data Availability

The datasets used and/or analyzed during the current study are available as MS Excel files (.xlsx) from the corresponding author upon reasonable request.

## Abbreviations

EEG: Electroencephalograms
BIS: bispectral index
SQI: signal quality index
EMG: electromyography
LOC: eyelash reflex is lost
ASA: American Society of Anesthesiologists
SPSS: Statistical Package for Social Sciences
SD: standard deviations
